# Protective effect of urotensin II receptor antagonist urantide and exercise training on doxorubicin-induced cardiotoxicity

**DOI:** 10.1038/s41598-023-28437-y

**Published:** 2023-01-23

**Authors:** Jing Sun, Jiedong Zhou, Shimin Sun, Hui Lin, Hanlin Zhang, Zuoquan Zhong, Jufang Chi, HangYuan Guo

**Affiliations:** 1 Shaoxing People’s Hospital (Shaoxing Hospital, Zhejiang University School of Medicine), Shaoxing, China; 2 Medical College of Shaoxing University, Shaoxing, China

**Keywords:** Cell biology, Drug discovery, Physiology

## Abstract

Doxorubicin (DOX) has a wide antitumor spectrum, but its adverse cardiotoxicity may lead to heart failure. Urotensin II (UII) is the most potent vasoconstrictor in mammals. It plays a role by activating the UII receptor (UT), the orphan G protein-coupled receptor (GPR14), collectively referred to as the UII/UT system. In the new version of "Chinese expert consensus on cardiac rehabilitation of chronic heart failure," it is pointed out that exercise rehabilitation is the cornerstone of cardiac rehabilitation. In this study, in vitro and in vivo assessments were performed using DOX-treated H9C2 cells and rats. It was found that the UT antagonist Urantide and exercise training improved DOX-induced cardiac insufficiency, reduced DOX-induced cardiomyocyte apoptosis, improved the structural disorder of myocardial fibers, and inhibited DOX-induced myocardial fibrosis. Further studies showed that Urantide alleviated DOX-induced cardiotoxicity by downregulating the expression levels of the p38 mitogen-activated protein kinase signaling pathway.

## Introduction

According to statistics from the American Cancer Society, it is estimated that there will be approximately 1.92 million new cancer cases and about 610,000 cancer deaths in the United States in 2022^1^. Doxorubicin (DOX) has become the cornerstone of chemotherapy for various cancers; however, its severe cardiotoxicity limits its clinical application. Cytotoxic chemotherapy is considered to have a relatively narrow therapeutic window; outside the optimal treatment range, there is toxicity or a lack of efficacy^2^. The incidence of cardiomyopathy was 5%, 26%, and 48% when the cumulative dose of anthracycline reached 400 mg/m^2^, 550 mg/m^2^, and 700 mg/m^2^, respectively. Even at low cumulative exposure, subclinical cardiac insufficiency was observed^3^. The typical feature of chronic heart toxicity is left ventricular systolic dysfunction, which develops into dilated cardiomyopathy and chronic heart failure^4^. Among 2625 patients who received anthracycline therapy, the average follow-up time after chemotherapy was 5.2 years, and the incidence of cardiac toxicity was 9% (cardiac toxicity was defined as a decrease in left ventricular ejection fraction [LVEF] > 10% and < 50%)^5^. In another study, 76 patients were evaluated using cardiac magnetic resonance imaging 6 months before and after chemotherapy. LVEF and left ventricular mass decreased by 5% in the anthracycline group but did not in other chemotherapy groups^6^.

Cardiomyocytes are rich in mitochondria, which are the main subcellular targets of DOX and may be one of the reasons for the vulnerability of cardiomyocytes to injury^7^. DOX-induced mitochondrial DNA (mtDNA) damage has been detected in animal models and the heart tissues of DOX-treated patients^8,9^. And fluorescent DNA dyes have revealed that anthracyclines are embedded in nuclear DNA and mtDNA, suggesting that mtDNA might be a direct target of anthracyclines. When mtDNA combines with DOX, it can result in the suspension of the mitochondrial oxidative electron transport chain, energy reduction, and cell apoptosis^10^.

The earliest Urotensin II (UII), a cyclic polypeptide containing 12 amino acids, was isolated from the nervous system tail pituitary of *Gillichthys mirabilis*^11^. In 1998, Coulouarn first reported that human UII was a cyclic peptide with 11 amino acids, whereas frog UII had 13 amino acids, realizing a substantial leap from fish to amphibians and finally to mammals^12^. One year later, the DNA sequence encoding human UII was isolated, and it was confirmed that human UII was bound to G protein-coupled receptor (GPR14), so it was named urotensin receptor (UT). GPR14 mRNA is highly expressed in cardiovascular tissues and the pancreas, and it was also confirmed that the vasoconstrictive ability of UII was one order of magnitude stronger than endothelin-1, making human UII the most potent vasoconstrictor found in mammals^13^. Previous studies have confirmed that the UII content in healthy people is extremely low or even zero. However, in patients with end-stage congestive heart failure, the immunoreactivity of UII is very high and is negatively correlated with ejection fraction, indicating that this polypeptide plays an important role in cardiovascular pathophysiology^14^.

In the animal model of right heart failure induced by pulmonary hypertension, after treatment with the UT antagonist, Urantide, we observed that compared with the pulmonary hypertension group, the systolic pulmonary artery pressure and mean pulmonary artery pressure of the Urantide group were significantly decreased^15^. In atherosclerotic rats, after the administration of Urantide, myocardial injury can be alleviated by blocking the UII/UT system and regulating the mitogen-activated protein kinase (MAPK) signaling pathway, reducing serum creatine kinase (CK) and lactate dehydrogenase (LDH) levels, and downregulating the levels of UII and its receptor, p38, p-extracellular signal-regulated kinase (ERK), and p-c-Jun N-terminal kinase (JNK) in myocardial tissue^16^. MAPK is widely distributed in the cells of various organisms, and its signaling pathway is abnormally activated in myocardial injury. Therefore, inhibiting the activation of the MAPK signaling pathway may be important for the repair of myocardial injury.

Center-based cardiac rehabilitation, defined by the American Heart Association, consists of five core components: exercise training, patient evaluation, risk factor management (smoking, blood lipids, blood pressure, body weight, and diabetes), dietary counseling, and psychosocial intervention^17^. The intensity of exercise is gradual, and the long-term goal is generally 40 min of aerobic exercise daily, 5 days a week. Patients with heart failure completed a 10-min warm-up and a 10-min cool-down period before and after each aerobic exercise, respectively^18^. Any exercise produced by skeletal muscle can result in energy consumption higher than basal metabolic rate. Peak oxygen consumption of patients with heart failure increases with exercise training. This effect is believed to be mediated through a variety of pathways, including reversing ventricular remodeling, followed by improving myocardial contractility and compliance, and improving microvascular circulation^19^.

## Materials and methods

### Animals and treatments

**Figure 1:**
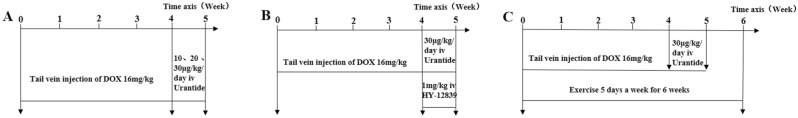
The study flow diagram for animal experiments. (**A**) The treatment groups were injected Urantide at 10, 20, and 30 μg/kg/day for 7 days consecutively. (**B**) The treatment groups were injected Urantide and (or) p38 MAPK-IN-1. (**C**) The treatment groups were injected Urantide and (or) exercise.

All experiments were performed per the Guide for the Care and Use of Laboratory Animals and were approved by the Ethics Committee of Experimental Research in Shaoxing People's Hospital. Six-week-old specific pathogen-free male Wistar rats, weighing approximately 200 g, were allowed free access to food and water at the Animal Experimental Center of Shaoxing People's Hospital and maintained at 25 °C under a 12 h light /12 h dark cycle. After 1 week of adaptive feeding, the rats were randomly divided into five groups (six rats in each group), and the control (CON) group was injected with an equal volume of normal saline. In the DOX group, DOX (Cat: HY-15142A, MedChemExpress) was injected into the tail vein once weekly for 4 weeks at a cumulative dose of 16 mg/kg^20^. The treatment groups were divided into three groups: Urantide (CAS:669089-53-6, Top Science) was injected at 10, 20, and 30 μg/kg/day through the tail vein for 7 days consecutively^21,22^ (Fig. 1A). Twenty-four rats were randomly divided into four groups: CON, DOX, DOX + Urantide (30 μg/kg/day for iv, 7 days), and DOX + Urantide + p38 MAPK-IN-1 (1 mg/kg for iv, the p38 MAPK inhibitor, HY-12839, MCE) to confirm whether p38 MAPK inhibition contributes to urantide-induced protection^23^ (Fig. 1B). Finally, the rats were anesthetized by sevoflurane inhalation (RWD Life Science Co., LTD, Guangdong, China).

After considering the importance and necessity of exercise rehabilitation in heart failure^24,25^, the rats were randomly divided into five groups after adaptive feeding for 1 week (6 rats in each group): CON, DOX, DOX + Exercise (swim once daily, 5 days a week for 6 weeks), DOX + Urantide (administered by tail vein injection at 30 μg/kg every time for 7 consecutive days), and DOX + Urantide + Exercise groups. In the first week, rats received adaptive training, which was gradually increased from 10 to 40 min, and the training time of 40 min was maintained every time from the second week)^26^ (Fig. 1C).

### Transthoracic echocardiography

LVEF and left ventricular fractional shortening (LVFS) were measured using the Philips iE33 system (Philips Medical, Best, the Netherlands) and an s5-1 probe (12–14 MHz), and transthoracic echocardiography images were obtained at the same time. Data analysis was performed using the Philips QLab 9 post-processing software.

### Histological assessment of myocardial injury

Following echocardiography, all rats were anesthetized, and their hearts were removed, rapidly weighed, photographed, and immediately frozen in liquid nitrogen until analysis. Part of the heart was fixed with 4% formalin, embedded in paraffin, and serially sectioned at 5 μm thickness. Hematoxylin and eosin (HE) staining was used to evaluate the morphology of the myocardium, and Masson staining was used for collagen analysis. All the sections were imaged using a Leica DM3000 biological microscope (Leica, Wetzlar, Germany) at 200 × magnification. Quantization was performed using ImageJ software (National Institutes of Health).

### Serological evaluation of myocardial injury

While removing the heart, a blood sample was taken from the tail vein to the procoagulant and centrifuged at 3500 rpm for 10 min after stewing. The supernatant was stored at − 80 °C. LDH (Nanjing Jiancheng Biological Engineering Research Institute) and CK isoenzyme (CK-MB, Nanjing Jiancheng Biological Engineering Research Institute) were detected using enzyme-linked immunosorbent assay kits.

### Transmission electron microscopy

The left ventricular tissues of the rats were fixed overnight with 2.5% glutaraldehyde, washed with buffer solution, and then fixed in 1% osmic acid. After thorough washing, a series of dehydration steps were performed. Finally, the tissues were embedded in Araldite for coronal sectioning and stained with toluidine blue. Changes in the mitochondria and muscle fibers were observed under a Cs-corrected transmission electron microscope (Titan G2 60-300, FEI, Hillsboro).

### Wheat germ agglutinin (WGA) staining

After dewaxing the 5 μm thick paraffin sections of the rat heart, the samples were incubated with 5 μg/mL WGA (L4895; Sigma-Aldrich) in the dark at 37 °C for 20 min. After washing thrice with phosphate-buffered saline, 4′,6-diamidino-2-phenylindole (DAPI, P36941; Invitrogen) was stained in the dark at 25 °C for 5 min. Images were acquired using a Nikon Ti-U fluorescence microscope (Minato-ku, Tokyo, Japan) and were observed at 100-fold magnification. The size of cardiac myocytes was determined by dividing the total area by the number of cardiac myocytes.

### Apoptosis assay

Paraffin sections of heart tissue were dewaxed with xylene and rehydrated in a fractionated ethanol series. The sections were incubated with protease K for 20 min at 37 °C, and the tissue was incubated with a TdT-mediated dUTP nick end labeling (TUNEL, Roche) reaction mixture for 1 h in the dark at 37 °C. DAPI (P36941; Invitrogen) was stained in the dark at 25 °C for 5 min, and the TUNEL-positive cells were observed under a Nikon Ti-U fluorescence microscope at a magnification of 400.

### Cell culture and treatment

H9C2 cells were obtained from the Cell Bank of the Chinese Academy of Sciences (Shanghai, China) and cultured in Dulbecco's modified Eagle's medium supplemented with 10% fetal bovine serum (Gibco, Grand Island, NY) and antibiotics (100 U/mL penicillin and 100 μg/mL streptomycin). Cells were maintained at 37 °C in a humidified incubator with 5% CO_2_. The cells were treated with 5 μmol/L DOX for 24 h to establish a toxic cell model^27^. The cells were treat with 10 nmol/L, 100 nmol/L, 1000 nmol/L Urantide for 24 h. Subsequently, the cells were divided into five groups again as follows: control group, DOX group, DOX + 20 nmol/L p38 MAPK-IN-1 (HY-12839, MCE), DOX + 1000 nmol/L Urantide, and DOX + Urantide + p38 MAPK-IN-1 for 24 h^28,29^. Finally, primary cardiomyocytes were extracted from the hearts of neonatal Wistar rats for culture. Similar to the H9C2 cells, they were firstly divided into five groups according to Urantide concentration, and then divided into five groups according to the inhibitor. To evaluate the effect of Urantide on DOX-induced apoptosis in breast cancer cells, they were cultured in RPMI 1640 medium (Gibco, Grand Island, NY) into three groups: the control group, the DOX group, and the DOX + 1000 nmol/L Urantide group.

### Assessment of mitochondrial membrane potential

H9C2 cells were incubated with 2.5 mmol/L JC‐1 dye (Solarbio, Beijing, China) in the dark at 37 °C for 30 min. Red JC‐1 aggregates represent normal hyperpolarized membrane potential, whereas green JC‐1 monomers represent mitochondrial membrane potential loss. Images were obtained using a fluorescence microscope at 400 × magnification.

### CCK-8 assay

Cell viability was analysed by Cell Counting Kit‐8 (CCK8, Beyotime, Shanghai, China) according to the manufacturer's protocols. Cells were seeded and cultured at a density of 8 × 10^3^/well in 100 μL of medium into 96‐well microplates (Corning, USA) for 24 h. Then, the cells were treated with DOX or 1000 nmol/L Urantide for 24 h, 10 μL of CCK‐8 reagent was added to each well and then cultured for 2 h. All experiments were performed in triplicate. The absorbance was analysed at 450 nm using a microplate reader (Bio‐Rad, Hercules, CA, USA) using wells without cells as blanks.

### Western blot analysis

Total protein was extracted from frozen tissues or H9C2 cells using radioimmunoprecipitation assay lysis buffer (Beyotime, China) containing phosphatase inhibitor cocktail II (MedChem Express, China). Proteins were separated by Sodium dodecyl-sulfate polyacrylamide gel electrophoresis and electroblotted to polyvinylidene difluoride membranes (Millipore, Billerica, MA, USA). Membranes were blocked with 5% skim milk for 1 h at 25 °C and hybridized with primary antibodies against B-cell lymphoma 2 (Bcl-2, ab32124, 1:1000), Bcl-2 Associated X-protein (Bax, ab32503, 1:1000), p38 (ab170099, 1:1000), and glyceraldehyde 3-phosphate dehydrogenase (ab8245, 1:1000) at 4 °C overnight. The next day, the membrane was treated with Horseradish peroxidase-labeled goat anti-rabbit or anti-mouse secondary antibodies (A0208, A0216, 1:1000, Beyotime, China), incubated at 25 °C for l h, and visualized using an excellent chemiluminescent substrate detection kit (Cat:32106, Pierce™ ECL Western Blotting Substrate, Thermo Scientific). Finally, the ImageJ software (National Institutes of Health) was used for quantification. The original images are shown in the supplementary material (Figs. S1–S23).

### Statistical analysis

For any multiple-group comparisons, a one-way analysis of variance was performed, and the results were presented as mean ± standard deviation. Data were analyzed using SPSS version 20.0 software (SPSS Inc, Chicago, IL, USA). Statistical significance was set at *P* < 0.05.

### Ethical statement

The study is reported in accordance with ARRIVE guidelines.

## Results

### Urantide improves DOX-induced cardiac damage in vivo

**Figure 2:**
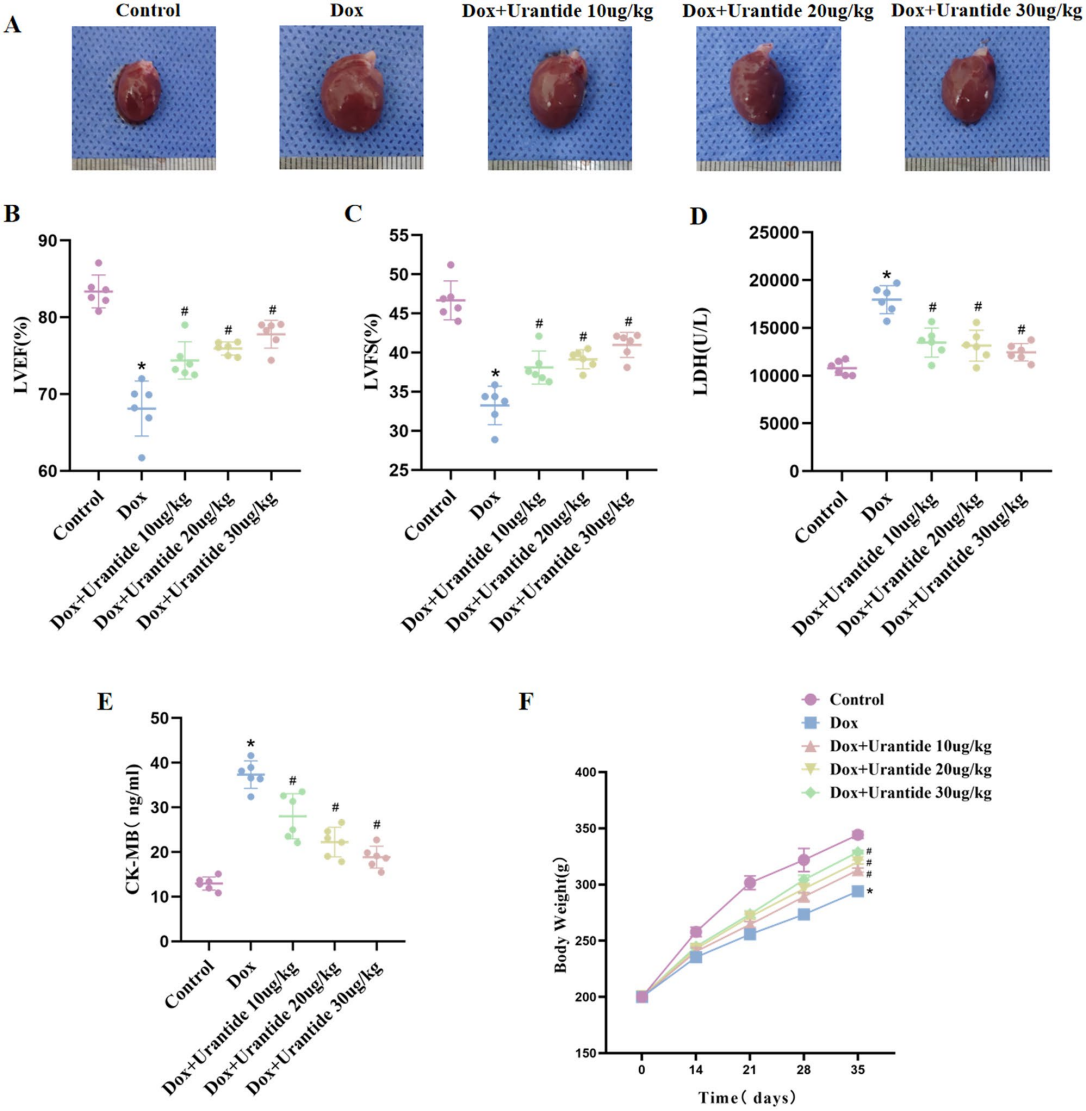
Urantide improves DOX-induced cardiac damage in vivo. (**A**) Representative images of global appearance of the heart in different groups. Results of LVEF (**B**) and LVFS (**C**) in the five groups (n = 6 per group). Enzyme‐linked immunosorbent assay was used on serum from rats to determine the expression of LDH (**D**) and CK-MB (**E**). (**F**) Average body weight change in 5 wk in rats from five groups (n = 6 per group). *P < 0.05, vs. the control; ^#^P < 0.05 vs. the DOX group.

**Figure 3:**
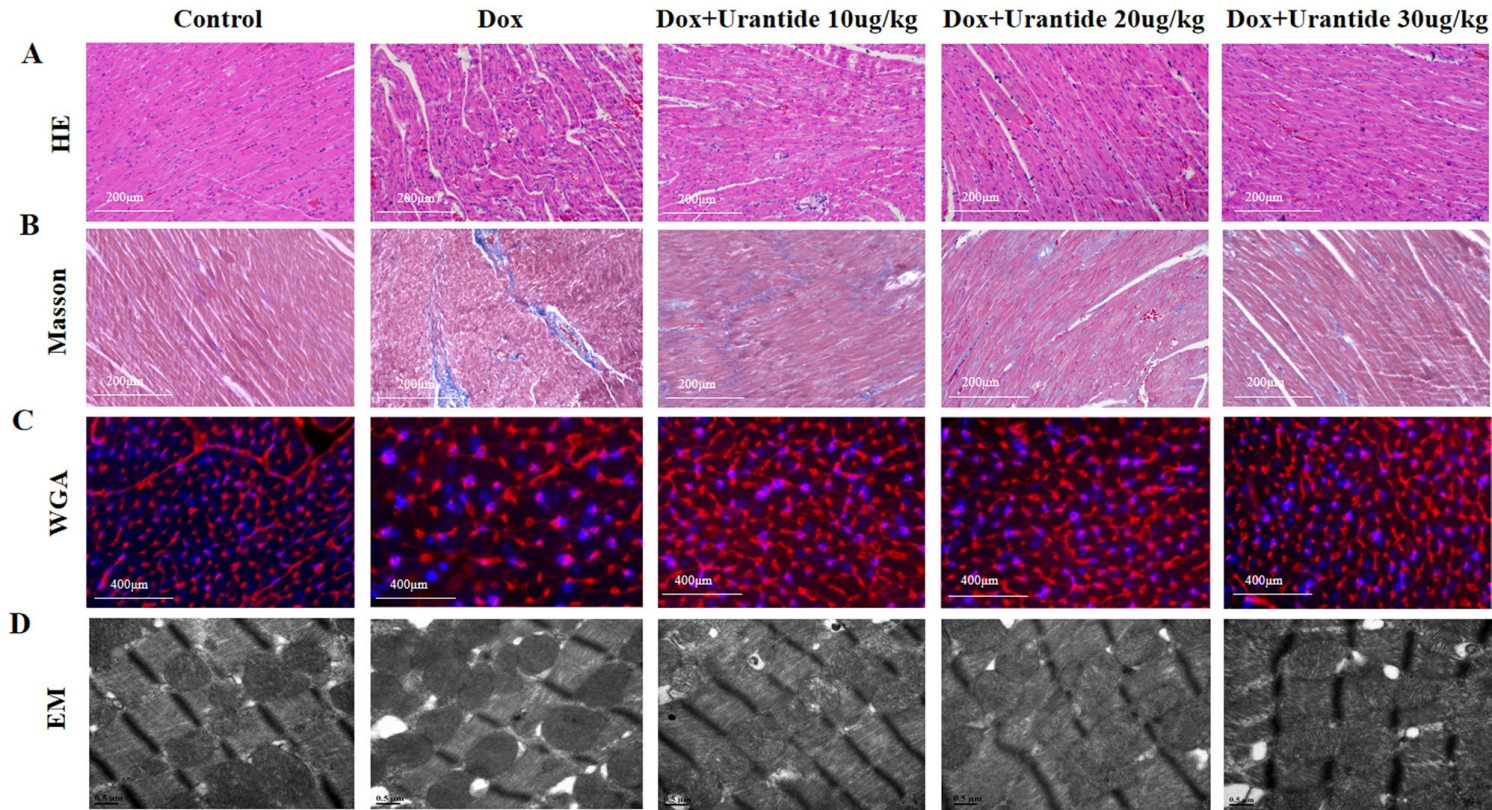
Urantide improves DOX-induced cardiac damage, myocardial fibrosis and cardiac myocyte hypertrophy in vivo. (**A**) HE staining of the heart tissue sections from Wistar rats treated. Bar = 200 μm. (**B**) Masson staining showing collagen content in the three Urantide treatment groups was decreased. (**C**) WGA staining of left ventricular tissue from rats for detect cardiac myocyte hypertrophy. Bar = 400 μm. (**D**) Transmission electron microscopy showing betterment of mitochondrial ultrastructure in Urantide group compared to DOX‐treated rats. Bar = 0.5 μm. (n = 6).

After the experiment, Fig. 2A shows that the rats in the DOX group had an enlarged heart, whereas the global appearance of the heart in the other four groups was normal. The LVEF (Fig. 2B) and LVFS (Fig. 2C) of the DOX group were significantly lower than those of the control group and increased after treatment with Urantide. The results of serological indicators of myocardial injury showed that after treatment with Urantide, LDH (Fig. 2D) and CK-MB (Fig. 2E) were significantly lower in the DOX group. As shown in Fig. 2F, the growth rate of body weight was significantly slower in the DOX group than in the control group. This trend was improved by intervention with Urantide. Compared with the CON group, HE staining in the DOX group demonstrated that the myofibers in the heart sections were obviously disordered, the normal appearance of myofibers was preserved and cell damage was significantly reduced after Urantide treatment (Fig. 3A). Compared with the CON group, Masson staining showed that the collagen content in the rat heart tissue of the DOX group was increased, while the collagen content in the three treatment groups was significantly decreased (Fig. 3B). In addition, we found that DOX-related cardiomyocytes were enlarged by WGA staining but decreased after Urantide treatment (Fig. 3C). As shown in Fig. 3D, the transmission electron microscope showed that compared with the CON group, the mitochondria of myocardial cells in the DOX group were swollen and deformed, mitochondrial cristae disappeared, and the arrangement of muscle fibers was disordered. Notably, the protective effect of Urantide on DOX-induced myocardial injury was more obvious with an increase in the dose.

### Urantide attenuates DOX-induced apoptosis in vivo

**Figure 4:**
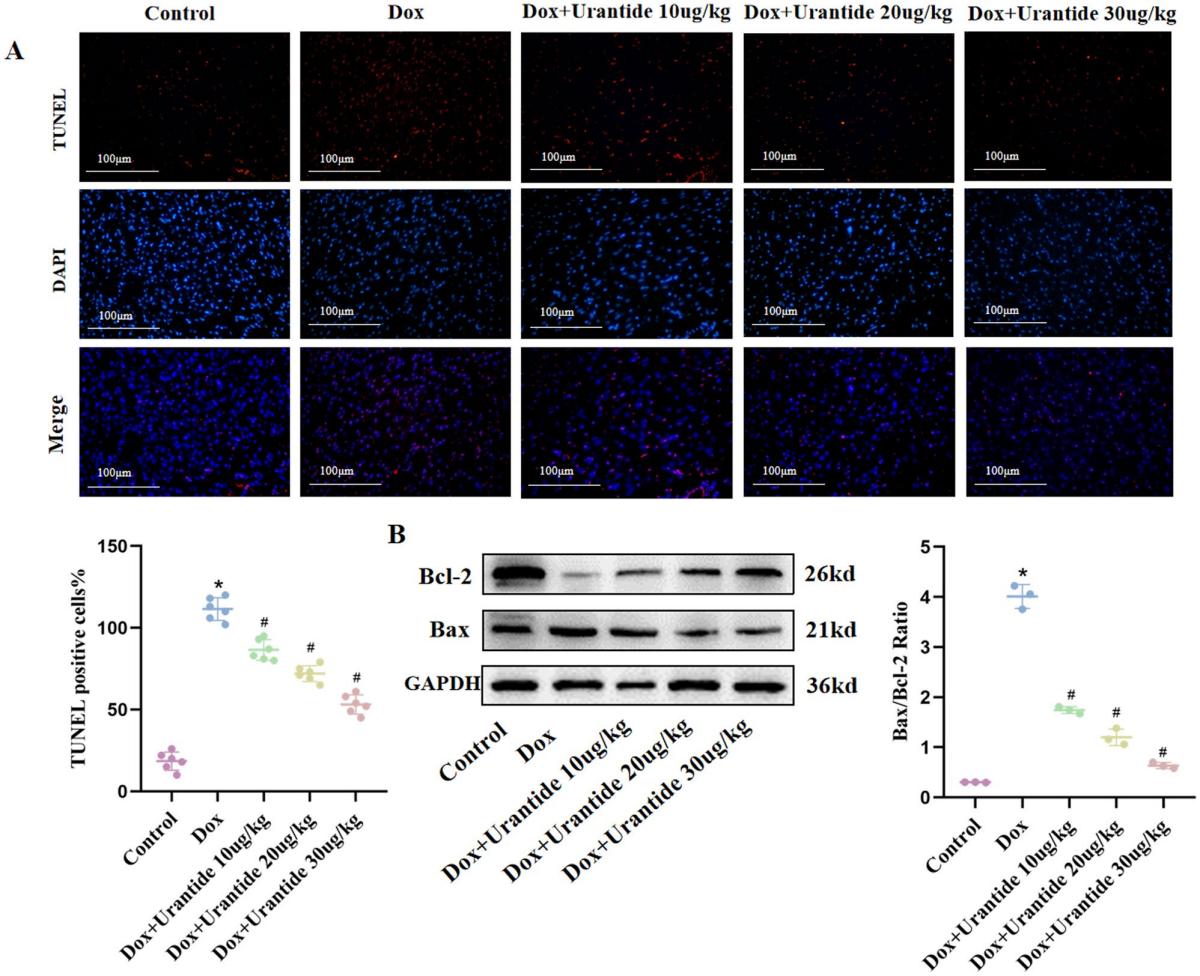
Urantide attenuates DOX-induced apoptosis in vivo. (**A**) Staining cells with red indicated TUNEL‐positive cells, DAPI staining (blue) indicated nucleus. Bar = 100 μm. Percentages of TUNEL‐positive cells of total cells were shown. (**B**) Protein expression of Bcl‐2 and Bax in the different treatment groups. (n = 6). *P < 0.05, vs. the control; ^#^P < 0.05 vs. the DOX group.

The correlation between apoptosis and DOX-induced cardiotoxicity has been proven in relevant studies; therefore, we explored the effect of Urantide in DOX-induced apoptosis. In TUNEL staining showed that the apoptosis rate of cardiomyocytes in the DOX group was significantly reduced after the treatment (Fig. 4A). Apoptosis-related proteins were subsequently detected, and DOX increased the expression of Bax, which was reversed by Urantide treatment (Fig. 4B, Figs. S1–S3 in supplementary material). These results indicate that Urantide could antagonize DOX-induced cardiomyocyte apoptosis, and the improvement was most significant in the 30 μg/kg treatment group.

### The attenuation of DOX-induced myocardial toxicity by Urantide was dependent on the downregulation of p38 in the MAPK pathway in vivo

**Figure 5:**
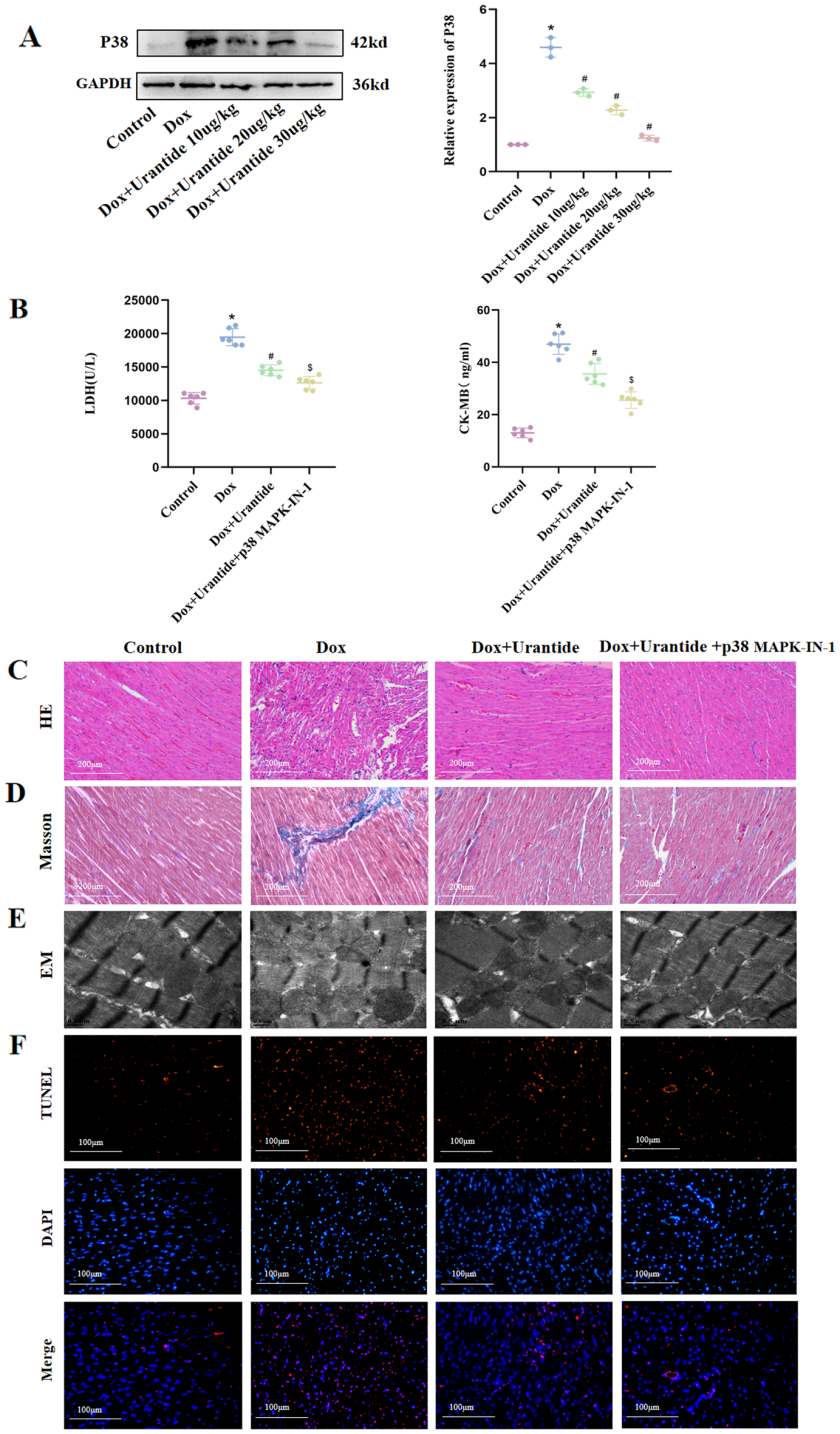

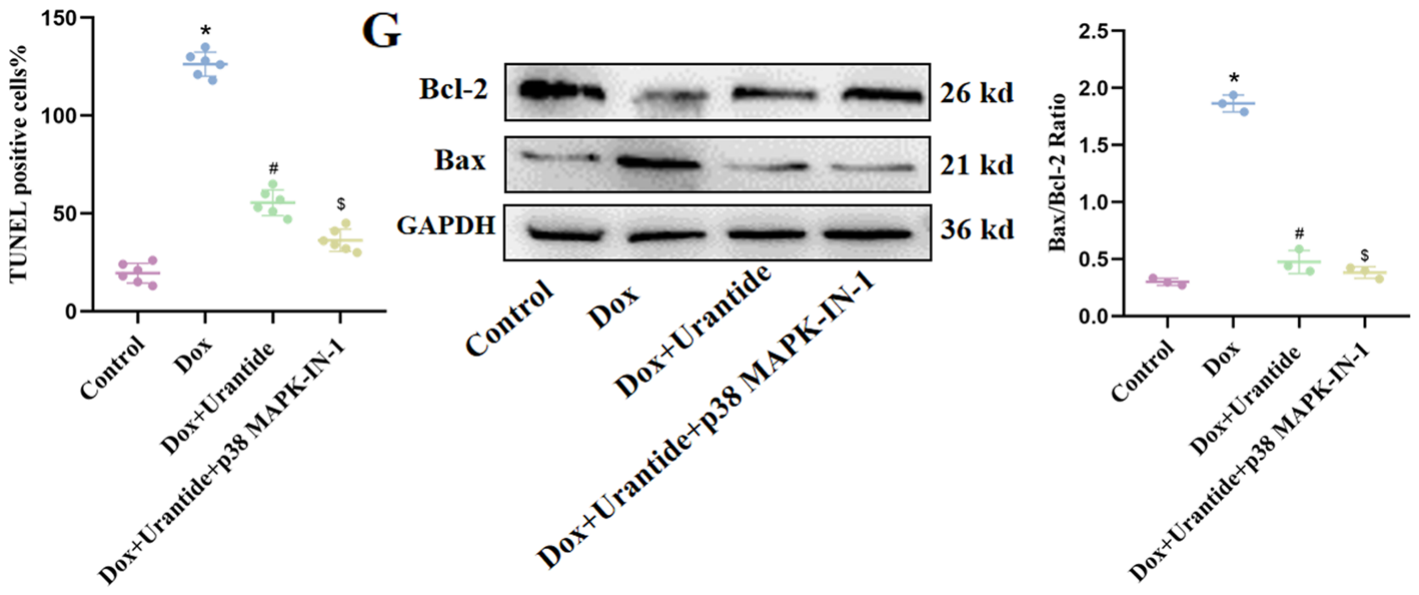
The attenuation of DOX-induced myocardial toxicity by Urantide was dependent on the downregulation of p38 in the MAPK pathway in vivo. (**A**) The expression level of the p38 MAPK pathway was downregulated in Urantide-treated. (**B**) CK-MB and LDH of the inhibitor group was decreased compared to Urantide‐treated rats. (**C**) Myocardial fiber appearance was more intense of the inhibitor group in HE staining. (**D**) Masson staining showed that the collagen content in the inhibitor group was significantly lower. (**E**) Transmission electron microscopy showing the beneficial of inhibitor. (**F**) Representative images of TUNEL staining of the heart tissues. (**G**) The levels of anti-apoptotic proteins were increased by the inhibitor. (n = 6). *P < 0.05, vs. the control; ^#^P < 0.05 vs. the DOX group; ^$^P < 0.05 vs. the Urantide group.

After Urantide intervention, compared to the DOX group, the expression level of the p38 MAPK pathway was significantly downregulated (Fig. 5A, Figs. S4, S5 in supplementary material). The p38 MAPK inhibitor revealed that the attenuation of DOX-induced myocardial toxicity by Urantide was dependent on the downregulation of the p38 MAPK pathway. Compared to the Urantide group, the myocardial injury index (CK-MB and LDH) of the inhibitor group was decreased (Fig. 5B), the disorder of myocardial fiber arrangement was reduced, and the appearance was more intense in HE staining (Fig. 5C). Masson staining showed that the collagen content of the heart tissue in the inhibitor group was significantly lower than that in the Urantide group (Fig. 5D). Transmission electron microscopy (Fig. 5E) showed that, compared with the Urantide group, the swelling of mitochondria in the inhibitor group was significantly reduced, the disappearance of local fusion of cristae was reduced, muscle fibers were arranged neatly, and the gap between myofibrils was shortened. TUNEL staining showed that apoptosis was significantly reduced after inhibitor treatment (Fig. 5F), and Western blot analysis showed that after the administration of Urantide, the levels of anti-apoptotic proteins were increased by the inhibitor (Fig. 5G, Figs. S6–S8 in supplementary material).

### Urantide attenuates DOX-induced apoptosis in vitro

**Figure 6:**
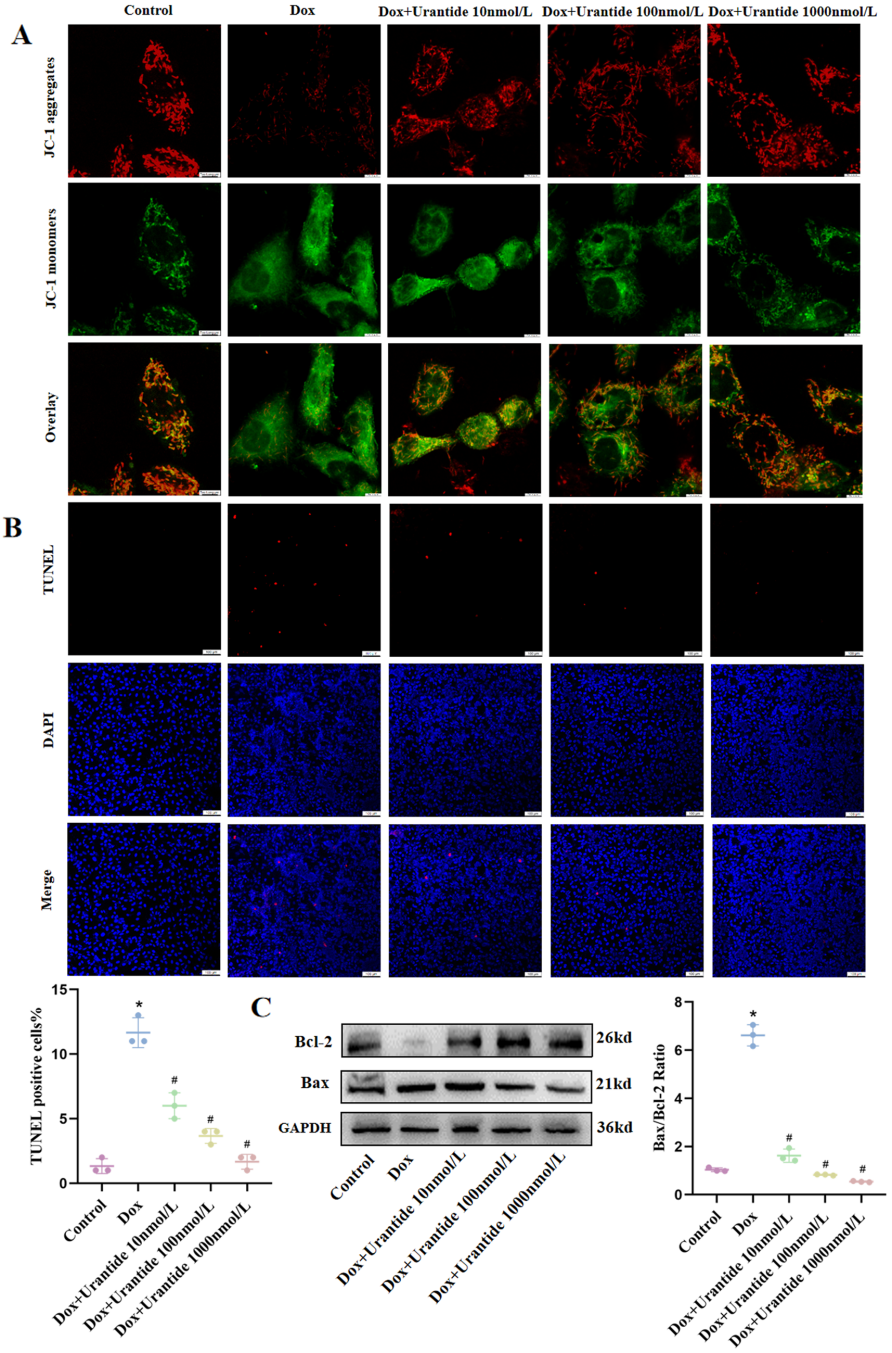
Urantide attenuates DOX-induced apoptosis in vitro. (**A**) Effects of Urantide (10 nmol/L, 100 nmol/L, 1000 nmol/L for 24 h) on DOX (5 μmol/L for 24 h)‐induced dissipation of mitochondrial membrane potential measured in H9C2 cells loaded with JC‐1 using fluorescence microscopy. (**B**) Representative images of TUNEL staining of H9C2 cells. (**C**) The relative expression of Bcl‐2 and Bax in H9C2 cells with or without Urantide treatment under DOX stimulation. *P < 0.05, vs. the control; ^#^P < 0.05 vs. the DOX.

To verify the effect of Urantide on apoptosis, we used DOX-induced H9C2 cells as the in vitro cell model. Determination of mitochondrial membrane potential by JC-1 method. Control cells expressed red JC-1 aggregates, representing a normal hyperpolarized membrane potential. After 24 h of DOX treatment, the cells exhibited green JC-1 monomers, and the mitochondrial membrane potential was lost. However, Urantide treatment resulted in orange fluorescence, indicating significant protection against DOX-induced mitochondrial membrane potential loss (Fig. 6A). After H9C2 cells were incubated with DOX for 24 h, their apoptotic rate increased significantly. However, Urantide treatment significantly inhibited the DOX-induced apoptosis (Fig. 6B). Furthermore, western blotting confirmed the same conclusion. (Fig. 6C, Figs. S9–S11 in supplementary material).

### The protective effect of Urantide on DOX-induced H9C2 cells in vitro depended on the downregulation of the p38 MAPK pathway

**Figure 7:**
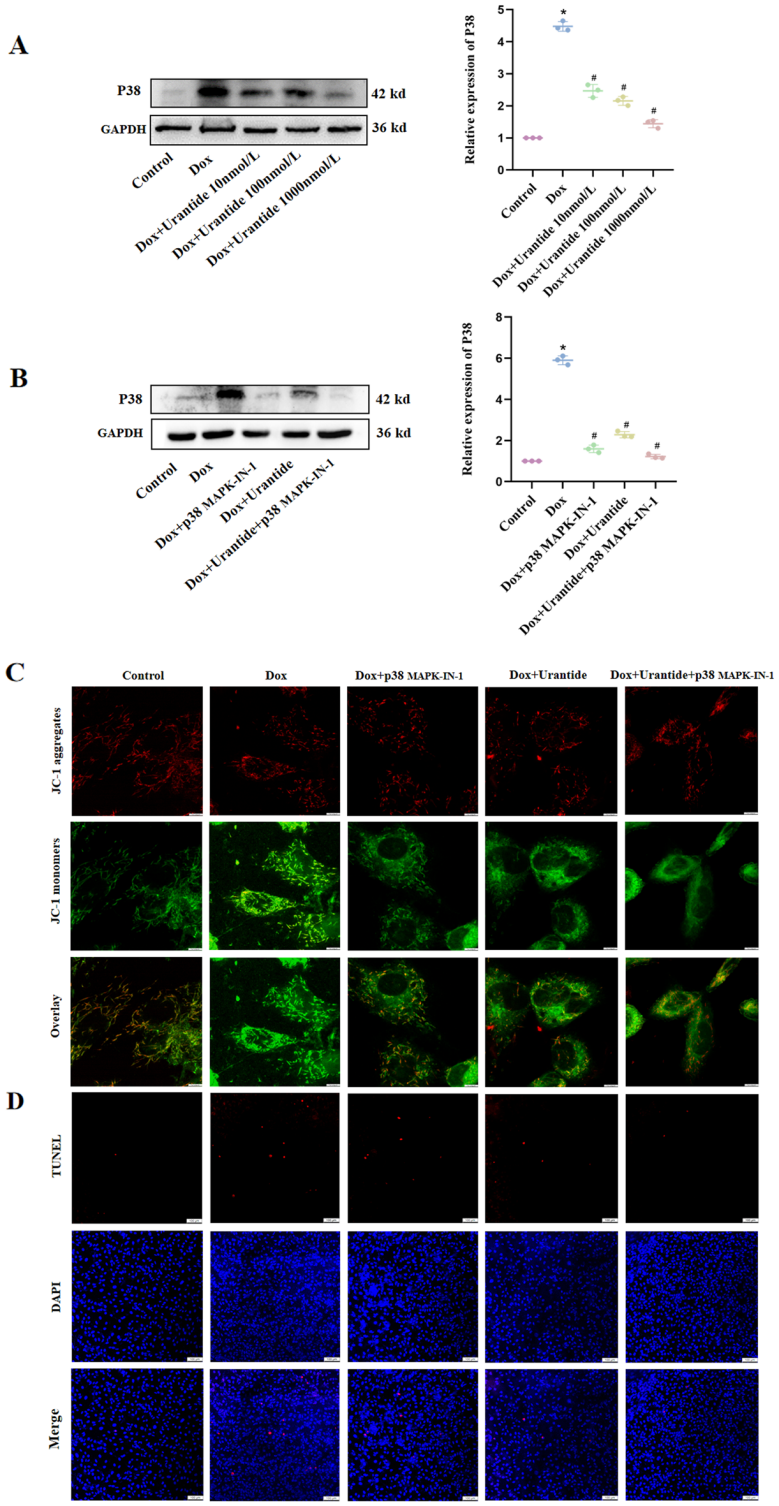

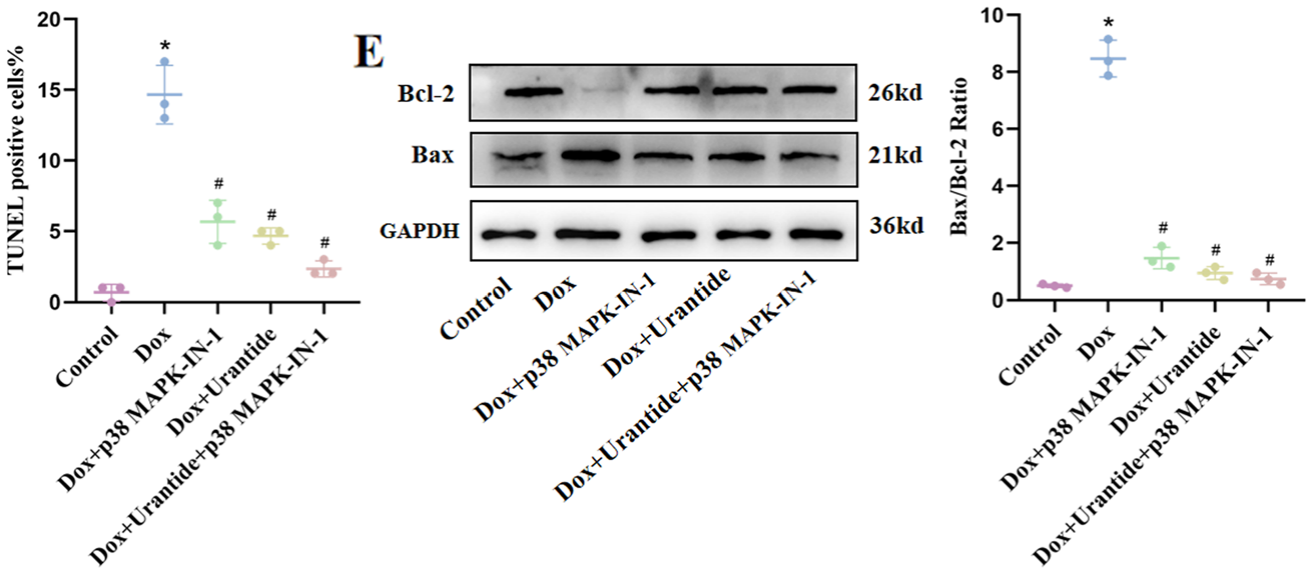
The protective effect of Urantide on DOX-induced H9C2 cells in vitro depended on the downregulation of the p38 MAPK pathway. (**A**) The expression level of the p38 MAPK pathway was downregulated in H9C2 cells with Urantide-treated compared to DOX group. (**B**) The expression level of the p38 MAPK pathway of the inhibitor group and Urantide group was decreased compared to DOX‐treated. (**C**) Determination of mitochondrial membrane potential by JC-1 method, the inhibitor group resulted in orange fluorescence. (**D**) Staining cells with red indicated TUNEL‐positive cells were obviously decreased in the inhibitor group and Urantide group. (**E**) The relative expression of Bcl‐2 and Bax in five groups. *P < 0.05, vs. the control; ^#^P < 0.05 vs. the DOX.

H9C2 cells were treated with p38 MAPK-IN-1 to confirm the role of the p38 MAPK pathway in the efficacy of Urantide in alleviating DOX-induced cardiotoxicity. We found that after the DOX treatment of H9C2 cells, the expression level of p38 MAPK significantly increased, and the Urantide treatment reversed this effect (Fig. 7A, Figs. S12, S13 in supplementary material). We subsequently found that p38 MAPK-IN-1 ± Urantide further downregulated p38 MAPK expression (Fig. 7B, Figs. S14, S15 in supplementary material) and reduced cells apoptosis (Fig. 7C–E, Figs. S16–S18 in supplementary material). These data suggest that Urantide mitigates DOX-induced cardiotoxicity by downregulating P38 MAPK expression in the MAPK signaling pathway.

### The protective effect of Urantide on DOX-induced primary cardiomyocytes depend on the down-regulation of the p38 MAPK pathway

**Figure 8:**
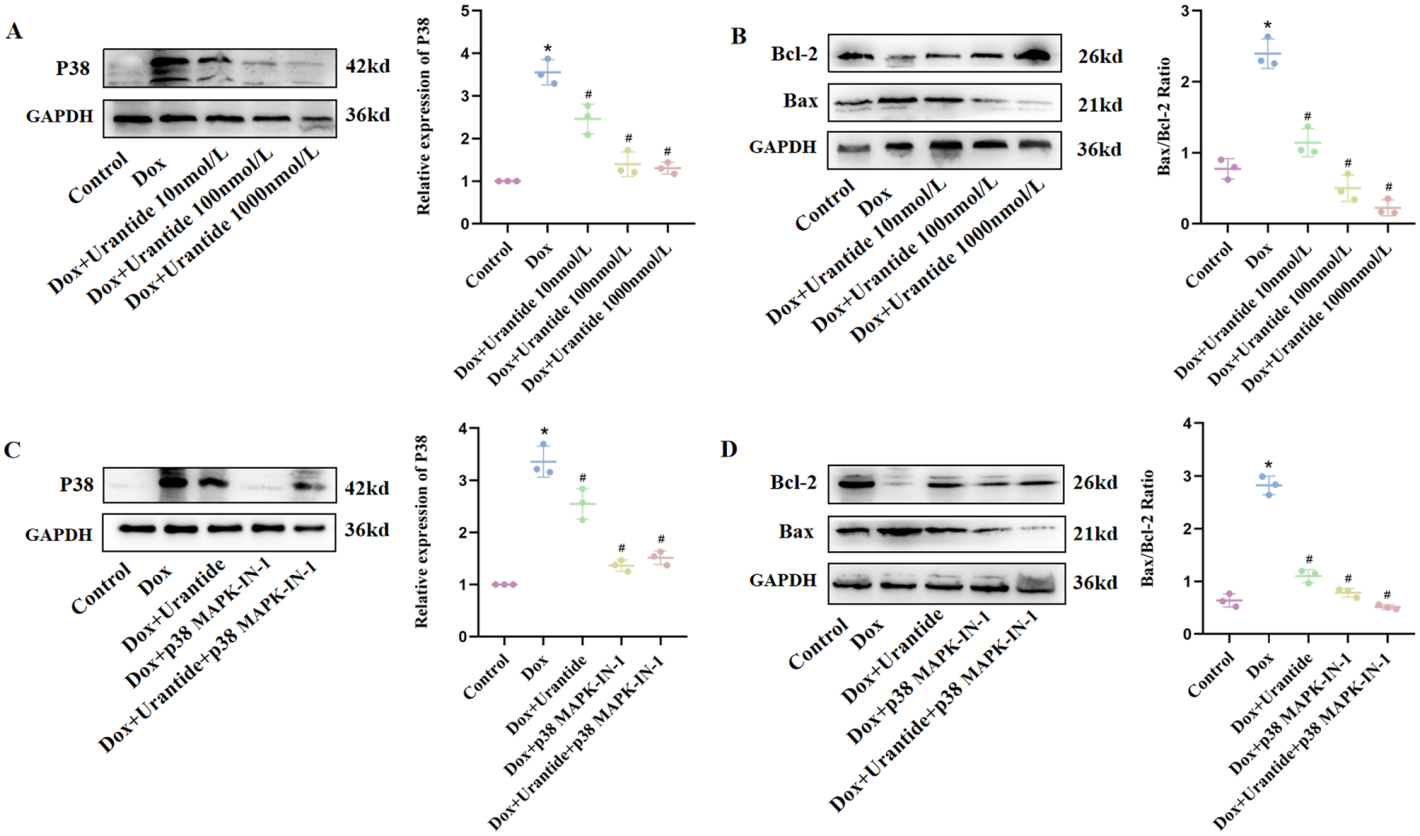
The protective effect of Urantide on DOX-induced primary cardiomyocytes depend on the down-regulation of the p38 MAPK pathway. (**A**,**C**) The expression level of the p38 MAPK pathway was downregulated in primary cardiomyocytes with Urantide or inhibitor-treated compared to DOX group. (**B**,**D**) The relative expression of Bcl‐2 and Bax in five groups. *P < 0.05, vs. the control; ^#^P < 0.05 vs. the DOX.

With the increase of Urantide concentration, compared with the DOX-induced primary cardiomyocytes, the Bax/Bcl-2 ratio and p38 MAPK expression level in the Urantide group were decreased gradually (Fig. 8A,B, Figs. S19–S23 in supplementary material). Subsequently, it was found that the addition of inhibitors could further down-regulate the expression of p38 MAPK and reduce primary cardiomyocytes apoptosis (Fig. 8C,D, Figs. S24–S28 in supplementary material). These data also justify our previous finding that Urantide reduced DOX-induced cardiotoxicity by down-regulating the P38 MAPK signaling pathway.

### Urantide and exercise alleviate DOX‐induced cardiac damage and apoptosis

**Figure 9:**
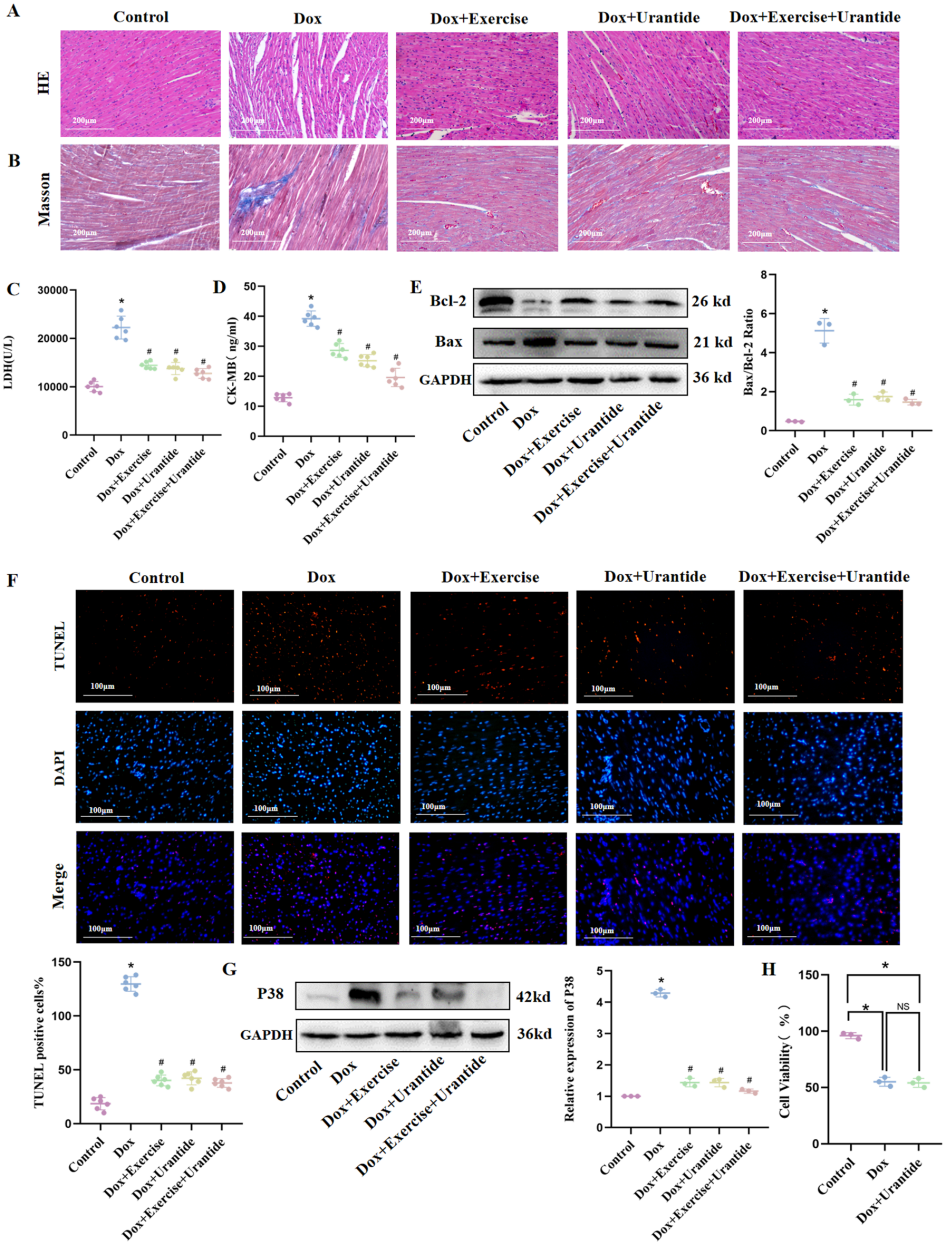
Urantide and (or) exercise alleviate DOX‐induced cardiac damage and apoptosis. (**A**) Myocardial fibers were arranged more neatly in the three treatment groups in HE staining. (**B**) Masson staining showed that the collagen content in the Urantide and (or) exercise group were significantly lower. Enzyme‐linked immunosorbent assay was used on serum from rats to determine the expression of LDH (**C**) and CK-MB (**D**). (**E**) DOX increased the Bax/Bcl-2 ratio, which was decreased by Urantide and (or) exercise treatments. (**F**) TUNEL-positive cells in the treatment group was significantly lower than in the DOX group. (**G**) The expression level of the p38 MAPK pathway was significantly downregulated by Urantide and (or) exercise. (**H**) The CCK8 was performed in breast cancer cells in vitro. *P < 0.05, vs. the control; ^#^P < 0.05 vs. the DOX. ^NS^P > 0.05 vs. the DOX.

Compared to the DOX group, the myocardial fibers were arranged more neatly in the three treatment groups (Fig. 9A), and the collagen content in the heart tissue was significantly reduced (Fig. 9B). LDH and CK-MB levels were significantly lower after Urantide administration and exercise than those in the DOX group (Fig. 9C,D). Moreover, DOX increased the Bax/Bcl-2 ratio, which was decreased by Urantide and exercise treatments (Fig. 9E, Figs. S29–S31 in supplementary material). TUNEL staining showed the same result: the number of positive cells in the treatment group was significantly lower than in the DOX group (Fig. 9F). The expression level of the p38 MAPK pathway was significantly downregulated by Urantide and exercise (Fig. 9G, Figs. S32, S33 in supplementary material).

### The effect of Urantide on DOX-induced apoptosis in breast cancer cells

The CCK8 experiment was performed in breast cancer cells. The results showed that compared with the control group, there was no significant difference in cell viability between the other two groups (Fig. 9H).

## Discussion

In this study, we demonstrated the protective effect of Urantide against DOX-induced myocardial toxicity in both in vivo and in vitro settings. Urantide reduced DOX-induced apoptosis in cardiomyocytes, upregulating the expression of Bcl-2 and downregulating Bax expression. Cardiac fibrosis, a common feature of many cardiac pathophysiological conditions, is related to cardiac dysfunction in DOX-induced cardiotoxicity. Collagen fibers were significantly deposited in DOX-treated rat hearts and significantly reduced after treatment with Urantide. P38 MAPK expression was upregulated in H9C2 cells and rats exposed to DOX, while Urantide downregulated p38 MAPK, and p38 MAPK inhibitors further improved DOX-induced myocardial toxicity. Our results showed that Urantide inhibited the DOX-induced p38 MAPK pathway, prevented DOX-induced cardiomyocyte apoptosis, and inhibited cardiac fibrosis progression in vivo. In DOX + exercise rats, DOX-induced myocardial injury and apoptosis were reversed, and exercise protected the heart to reduce DOX-induced cardiotoxicity. Simultaneously, we found that the p38 MAPK pathway was downregulated in the exercise group. Considering that H9C2 cells are recognized as a widely used cardiomyocyte line, which exhibit similar biological activity and metabolic characteristics to living cardiomyocytes^30,31^, but are different from the non-renewable reproduction characteristics of primary cardiomyocytes. Therefore, Western Blot was repeated in primary cardiomyocytes and the results were consistent with H9C2.

The anticancer activity of DOX is mainly exerted through DNA embedding and inhibition of topoisomerase II. However, DOX has dose-dependent cardiotoxicity, resulting in an increased risk of death, with various mechanisms of which myocardial injury and apoptosis have been widely reported^32,33^. It has been found that Berberine has a protective effect on DOX-induced heart injury and can significantly reduce and inhibit its oxidative stress and apoptosis^34^. Resveratrol can improve DOX-induced cardiac dysfunction by inhibiting myocardial fibrosis and reducing apoptosis^35^. Collagen levels were significantly increased in DOX-treated rats, indicating cardiac fibroblast activation and excessive extracellular matrix deposition. Following treatment with Urantide, we found that DOX-induced myocardial fibrosis and apoptosis were greatly improved, and the expression levels of apoptosis-related proteins were significantly affected, with upregulation of Bcl-2 and downregulation of Bax.

DOX can induce the AMP-activated protein kinase signaling pathway and activate AKT and MAPK through genotoxicity and oxidative stress^36,37^. The Urantide used in this study has been confirmed to provide cardiovascular protection in animal models of right heart failure and atherosclerosis caused by pulmonary hypertension. Notably, Urantide can also reduce myocardial damage in atherosclerotic rats by regulating p38 and ERK levels in the MAPK signaling pathway^15,16^. We tested the effect of Urantide on DOX-exposed H9C2 cells and p38 expression in rats. The p38 signaling pathway was activated after DOX stimulation, while Urantide inhibited the p38 pathway to alleviate DOX-induced myocardial injury. The results further demonstrated that p38 inhibitors could reverse DOX-induced cardiotoxicity and cardiac dysfunction.

Studies have confirmed that exercise can inhibit DOX-induced apoptosis and reduce tissue damage. It can inhibit DOX-induced oxidative damage by inhibiting the level of NADPH oxidase 2 but does not regulate other key antioxidant enzymes^38^. In DOX + exercise rats, ejection fraction and fractional shortening improved over time, indicating that exercise intervention could reduce myocardial toxicity following DOX induction and confirming that exercise reduced the sensitivity to myocardial injury^39^. Exercise inhibited indicators of cardiac injury, and increases in LDH and CK-MB levels generally represented their leakage into the circulation from damaged myocardial cell membranes, indicating that exercise helped ameliorate DOX-induced cardiotoxicity. Additionally, DOX-treated cardiomyocytes were attributed to the inhibition of AKT, which activates p38 MAPK and nuclear factor-κB pathways^40^.

Some studies have shown that the serum concentrations of atenolol, propranolol and doxycycline are increased due to exercise; Increased physical activity in patients taking warfarin has been shown to decrease the international normalized ratio^41^. In addition, some articles pointed out that the absorption of weakly basic drugs might be increased during exercise, and the passive paracellular permeability involved would allow for greater absorption^42^. Some researchers used rat exercise training to evaluate cardiac function and DOX accumulation. The results showed that 10 weeks of exercise pretreatment reduced the accumulation of myocardial DOX, and had effects on the pharmacokinetics of doxorubicin, which might affect its anti-cancer effect^43^. Of note, however, DOX chemotherapy is considered to have a relatively narrow therapeutic window, with an incidence of 5%, 26%, and 48% of cardiomyopathy at cumulative doses of 400, 550, and 400 mg/m^2^, respectively. Subclinical cardiac insufficiency was observed even at low cumulative exposures. In clinical practices, the pros and cons should be weighed before drug administration, and the drug dose should be carefully selected. Although exercise reduces the accumulation of DOX, it may be beneficial for DOX to be within its relatively narrow treatment window, demonstrating from the side that exercise can reduce the toxic effect of DOX to a certain extent.

In addition, treating rat neonatal cardiomyocytes with DOX (1 μM, 16 h) and urotensin II (10 nM or 100 nM), the results showed the anti-apoptotic effect of urotensin II^44^, which seemed to be in sharp contrast to our results. These contradictory results could be partially explained by the heterogeneity of urotensin II, and the indicated action direction might also be different due to the concentration and action time. We have found that 48 h after neonatal rat cardiomyocytes were treated with high concentration of urotensin II (500 nM), significant pyroptosis was induced, which is a form of programmed cell death. At the same time, as an antagonist of urotensin II receptor, Urantide eliminated urotensin II-induced pyroptosis^45^. Another researcher used DOX and urotensin II to treat human umbilical vein endothelial cells (HUVEC) to evaluate cell apoptosis, and the results showed that urotensin II protected HUVEC from DOX-induced apoptosis^46^. As mentioned above, due to the heterogeneity, urotensin II has different effects on different cells. For example, lipopolysaccharide (LPS)/d-galactosamine (GalN)-treated mice showed massive apoptosis of hepatocytes, significant inflammatory infiltration of the liver and significant increase in the expression of urotensin II and its receptors, which were significantly reduced by pretreatment with urotensin II receptor antagonist Urantide^47^.

The urotensin pathway of DOX-induced cardiotoxicity is greatly affected by environmental factors and external factors. For example, compared with cells only exposed to DOX, the co-incubation of bisphenol a(BPA) and DOX significantly increased cardiomyocytes apoptosis and increased the DOX-related cardiotoxicity. Notably, co-incubations of cells with DOX and BPA at 0.002, 0.02, 0.2, and 2 M resulted in approximately 50, 75, 85, and 100% lower IC50 values compared to cells exposed to DOX alone, indicating that BPA is also cardiotoxic at very low concentrations^48^. This may mean that the environment will produce errors in our experimental results, but our rats are housed in SPF grade animal laboratory, so this concern is negligible.

Earlier, our research team has conducted research on DOX-induced cardiomyocyte apoptosis, which is manifested as increased cell death and up-regulated expression levels of NLRP3, caspase-3, IL-1β, IL-18 and GMDSD-N, which are all key proteins representing cell apoptosis. Knockdown of TINCR reverses DOX-induced apoptosis in vitro and in vivo, and the mechanism suggests that TINCR enhances NLRP3 mRNA and thus increases NLRP3 levels by recruiting IGF-2 BP1. Inhibition of NLRP3 or IGF-2 BP1 attenuated the effects of TINCR on DOX-induced cardiotoxicity and focal death^49^. As mentioned above, high concentrations of urotensin II lead to pyroptosis in neonatal rats. As an antagonist of urotensin II receptor, Urantide alleviates this effect^45^, which is consistent with our conclusion that Urantide has a protective effect on DOX-induced myocardial toxicity.

However, our study had certain limitations. First, it is still uncertain whether Urantide can enhance the chemotherapy efficacy of DOX; however, current research has found that it can alleviate DOX-induced cardiac dysfunction. Second, whether exercise exerts a protective effect against DOX-induced myocardial toxicity by downregulating the p38 MAPK pathway. Furthermore, the results of DOX-induced cardiotoxicity in rats cannot be fully mapped to humans. Therefore, further verification is required in future clinical studies.

**Figure 10:**
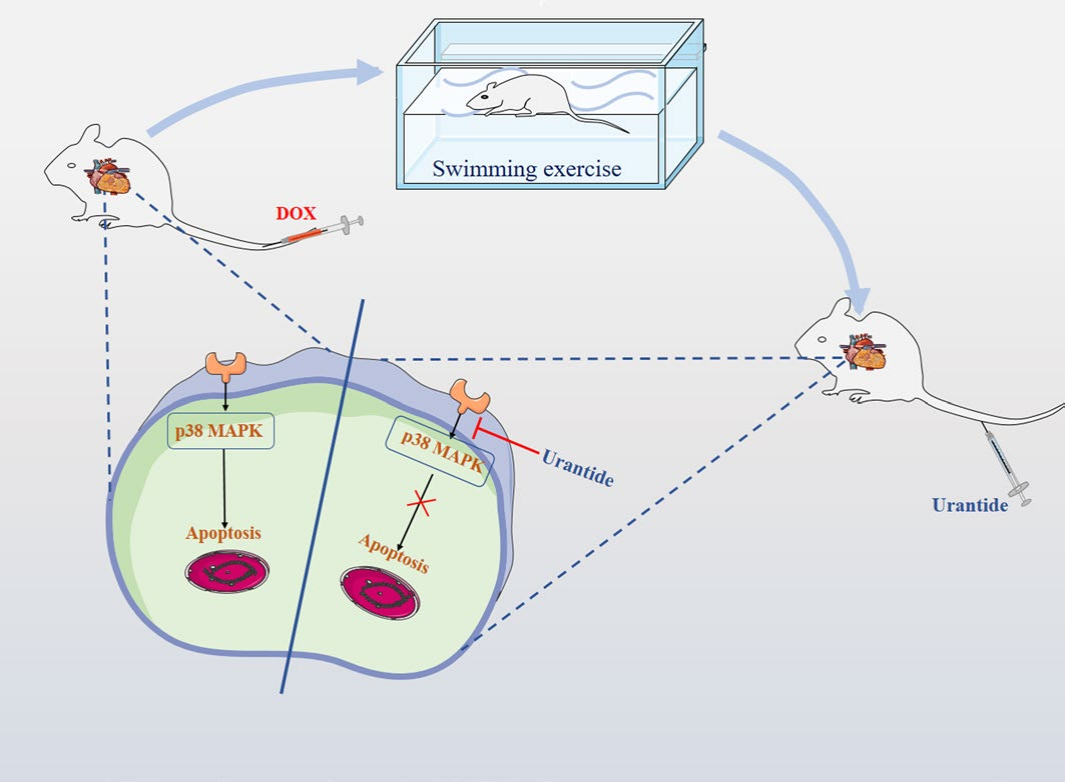
The expression level of the p38 MAPK pathway was upregulated in rats exposed to DOX, while Urantide and exercise were downregulated p38 MAPK and attenuated cardiac damage.

In conclusion, our data showed that Urantide and exercise could protect against DOX-induced cardiac fibrosis and apoptosis in rats, ultimately improving cardiac function and inhibiting DOX-stimulated apoptosis by downregulating the p38 MAPK pathway. These results suggest that Urantide and exercise may be promising new therapeutic strategies to alleviate DOX-induced cardiotoxicity (Fig. 10).

## Supplementary Information

Supplementary Figures.

## Data Availability

The data used to support the findings of this study are included in the article.
