# Supplementary material for: Protective effect of urotensin II receptor antagonist urantide and exercise training on doxorubicin-induced cardiotoxicity
Source: Sci Rep. 2023 Jan 23;13:1279. doi: 10.1038/s41598-023-28437-y (PMC9870887; doi:10.1038/s41598-023-28437-y)
Supplement: Supplementary file 1 — Supplementary Figures. [file 41598_2023_28437_MOESM1_ESM.docx]

Fig S1 Original image of Bcl-2 in FIGURE 4B


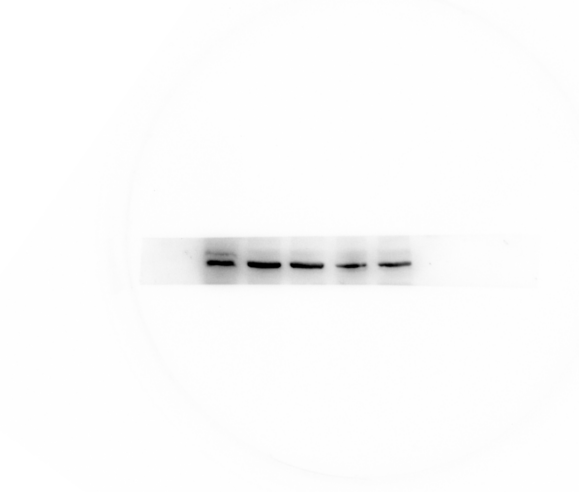


Fig S2 Original image of Bax in FIGURE 4B





Fig S3 Original image of GAPDH in FIGURE 4B


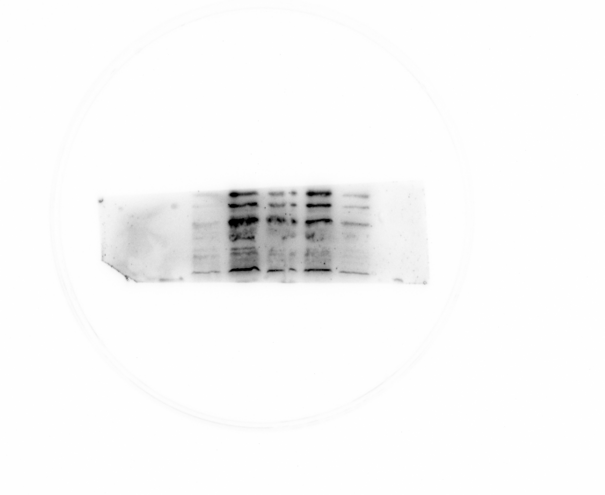


Fig S4 Original image of P38 in FIGURE 5A


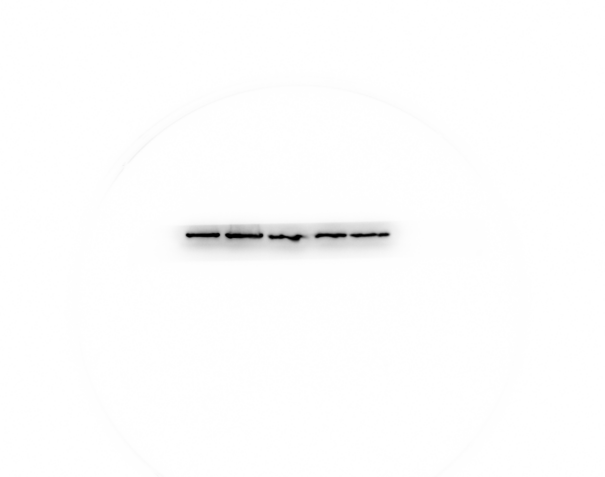


Fig S5 Original image of GAPDH in FIGURE 5A





Fig S6 Original image of Bcl-2 in FIGURE 5G





Fig S7 Original image of Bax in FIGURE 5G





Fig S8 Original image of GAPDH in FIGURE 5G


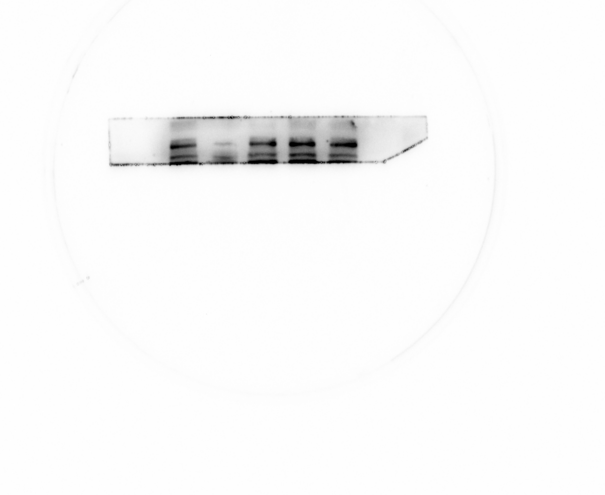


Fig S9 Original image of Bcl-2 in FIGURE 6C


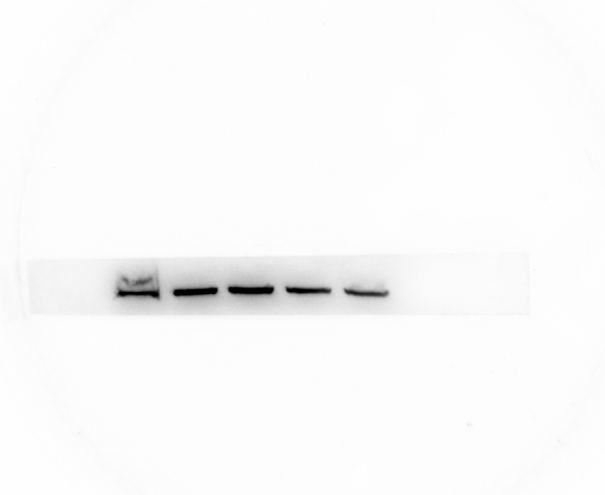


Fig S10 Original image of Bax in FIGURE 6C


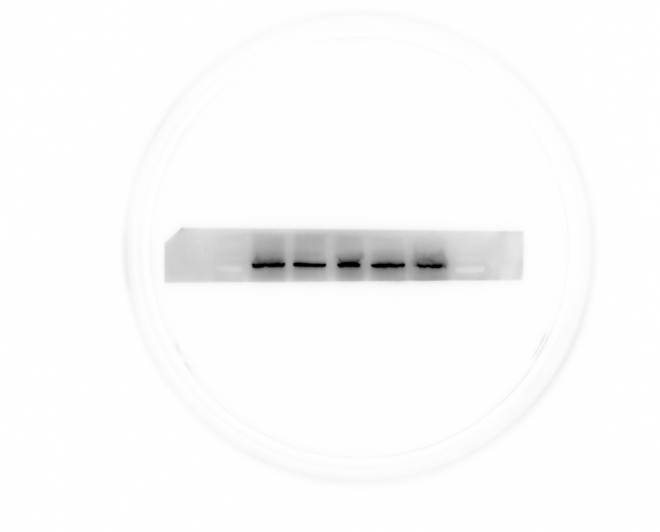


Fig S11 Original image of GAPDH in FIGURE 6C


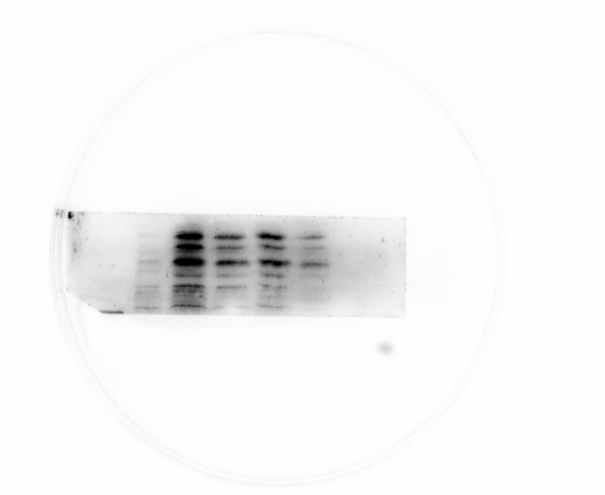


Fig S12 Original image of P38 in FIGURE 7A


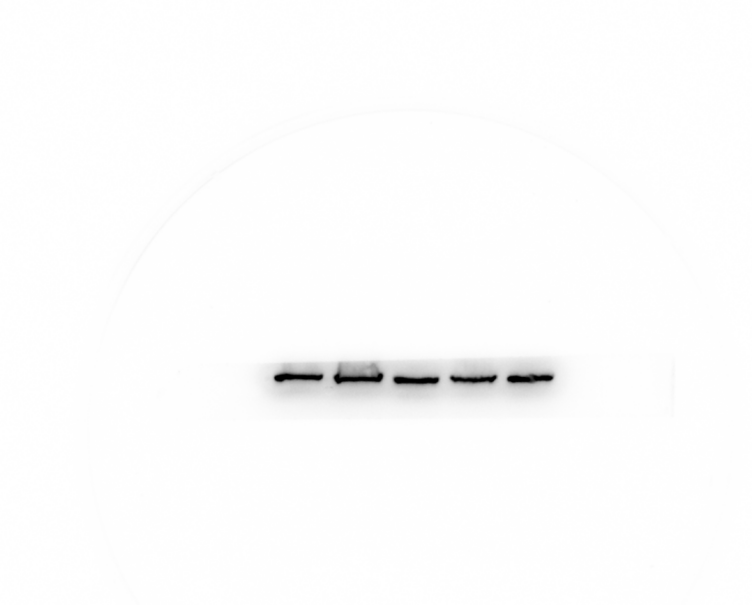


Fig S13 Original image of GAPDH in FIGURE 7A


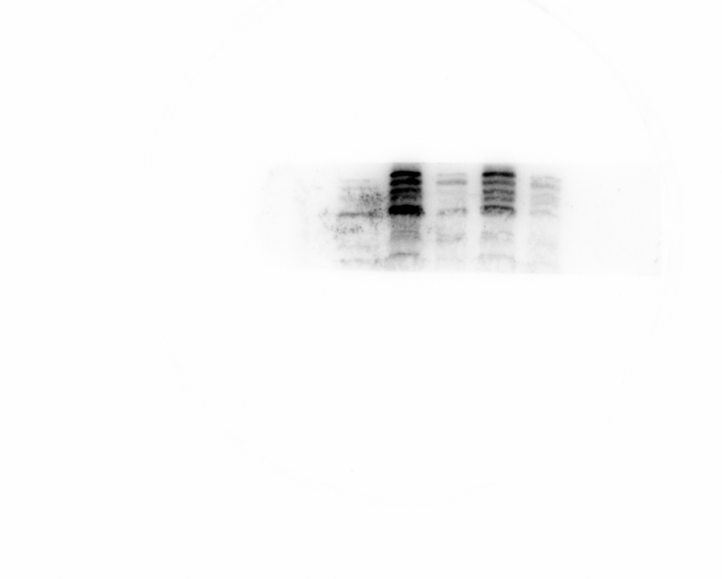


Fig S14 Original image of P38 in FIGURE 7B


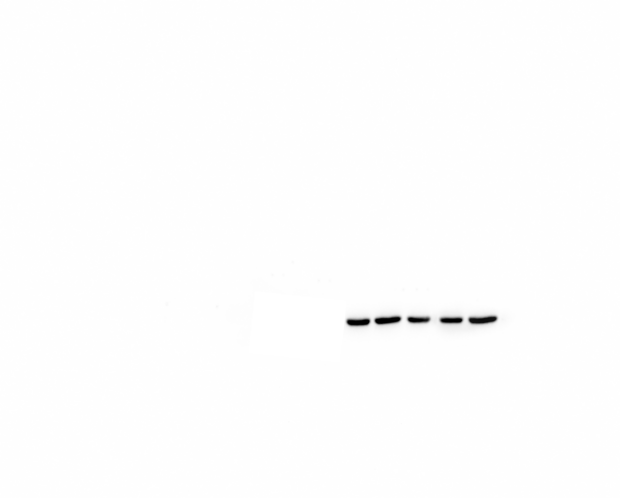


Fig S15 Original image of GAPDH in FIGURE 7B


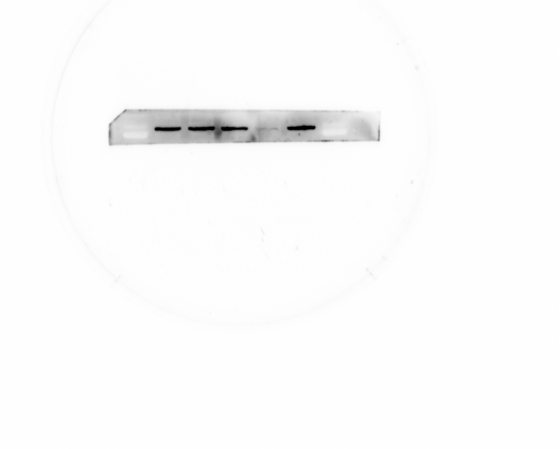


Fig S16 Original image of Bcl-2 in FIGURE 7E


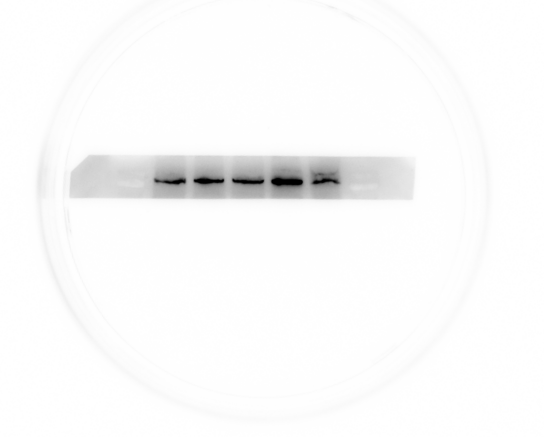


Fig S17 Original image of Bax in FIGURE 7E


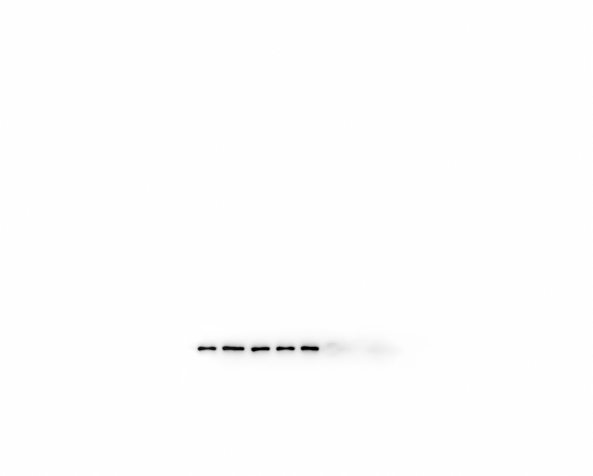


Fig S18 Original image of GAPDH in FIGURE 7E


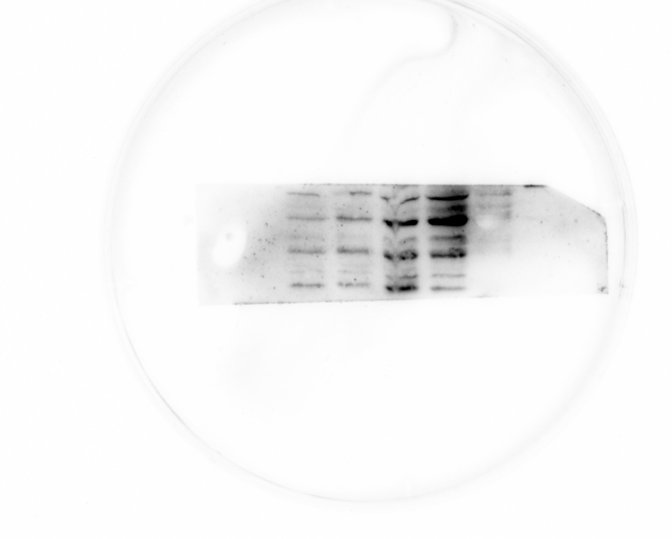


Fig S19 Original image of P38 in FIGURE 8A


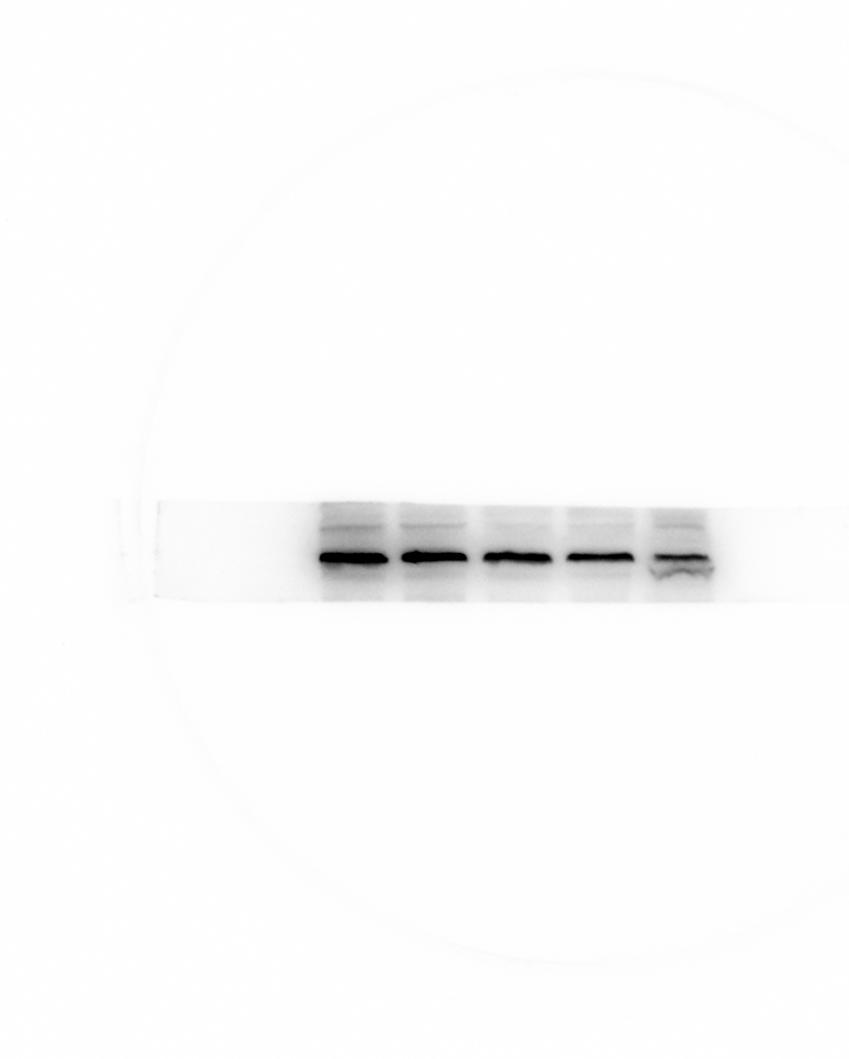


Fig S20 Original image of GAPDH in FIGURE 8A


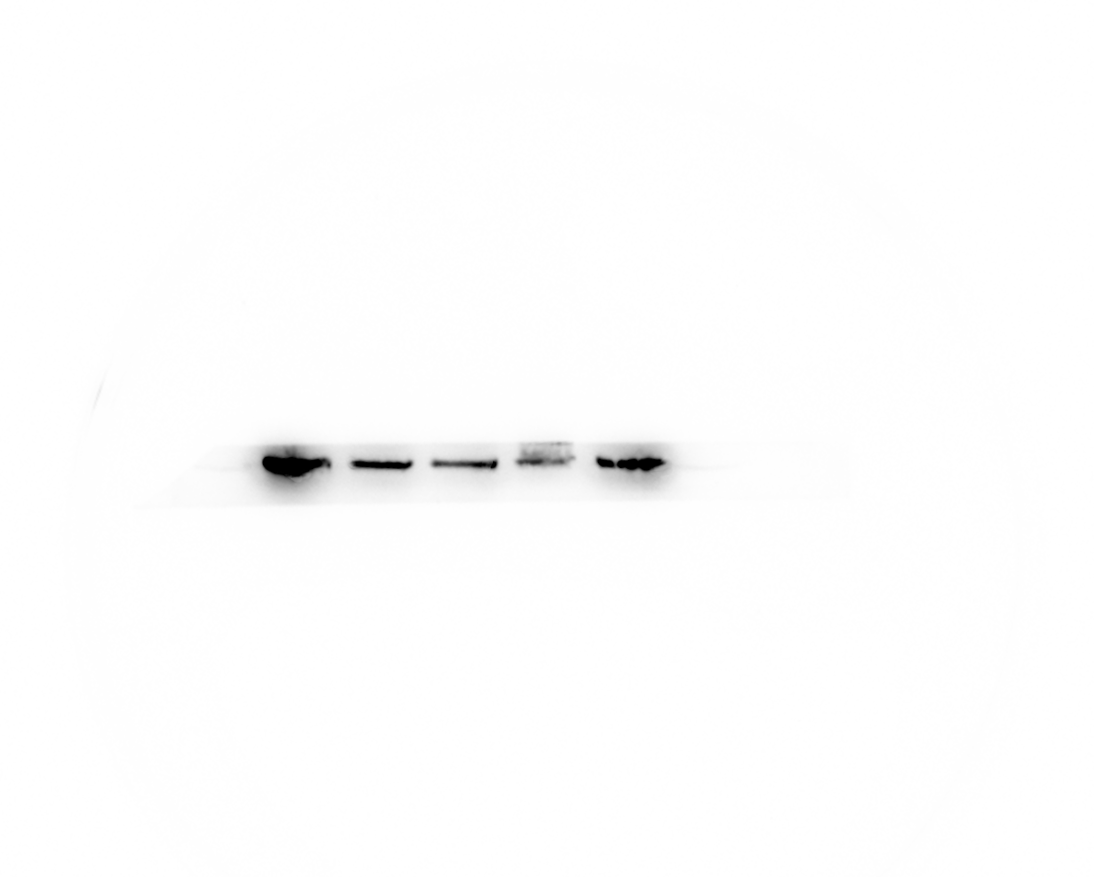


Fig S21 Original image of Bcl-2 in FIGURE 8B


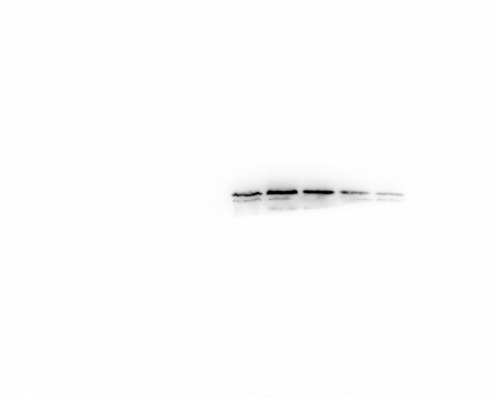


Fig S22 Original image of Bax in FIGURE 8B


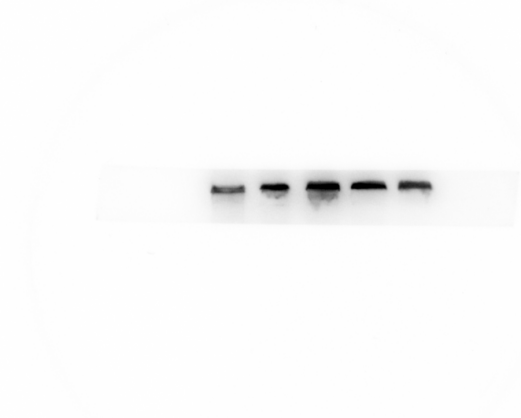


Fig S23 Original image of GAPDH in FIGURE 8B


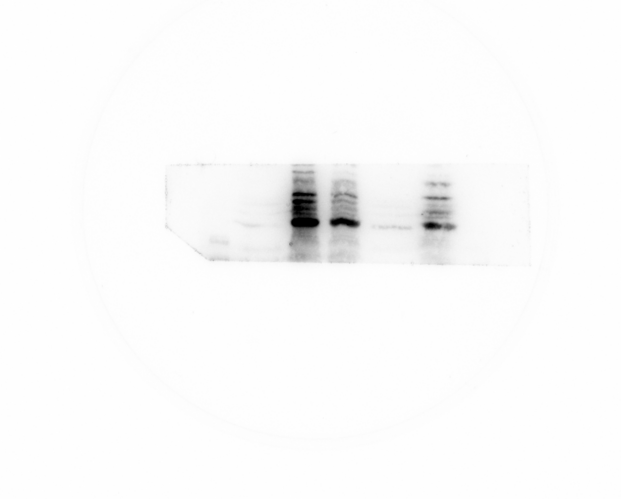


Fig S24 Original image of P38 in FIGURE 8C


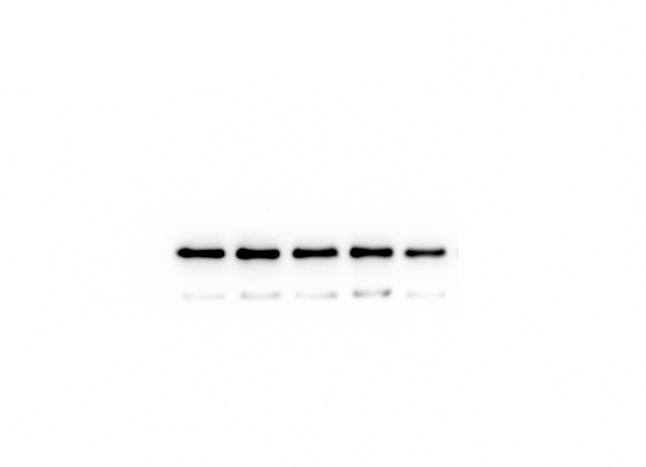


Fig S25 Original image of GAPDH in FIGURE 8C


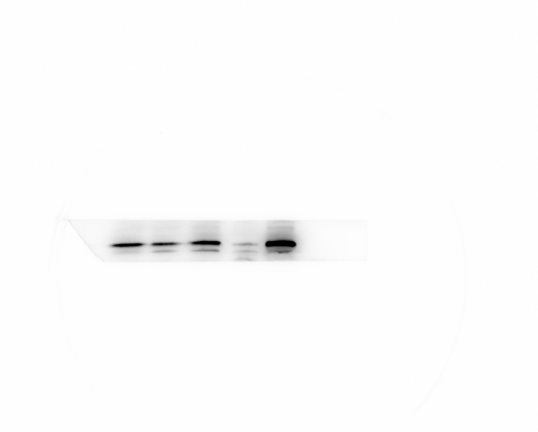


Fig S26 Original image of Bcl-2 in FIGURE 8D


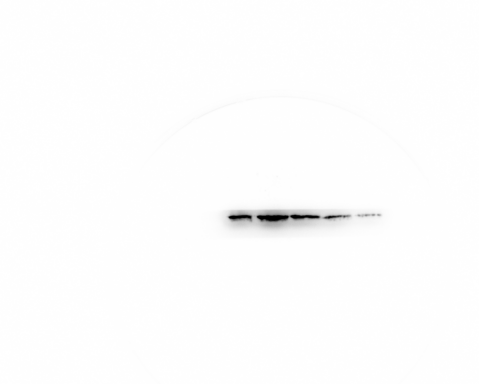


Fig S27 Original image of Bax in FIGURE 8D


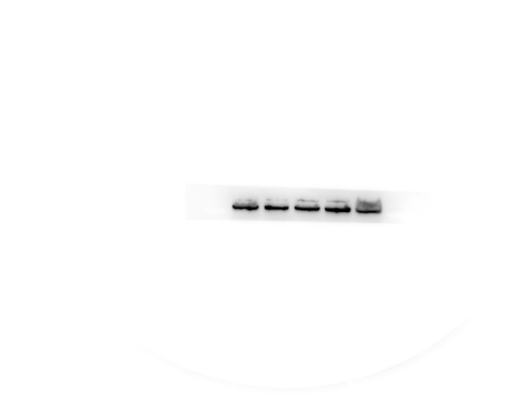


Fig S28 Original image of GAPDH in FIGURE 8D


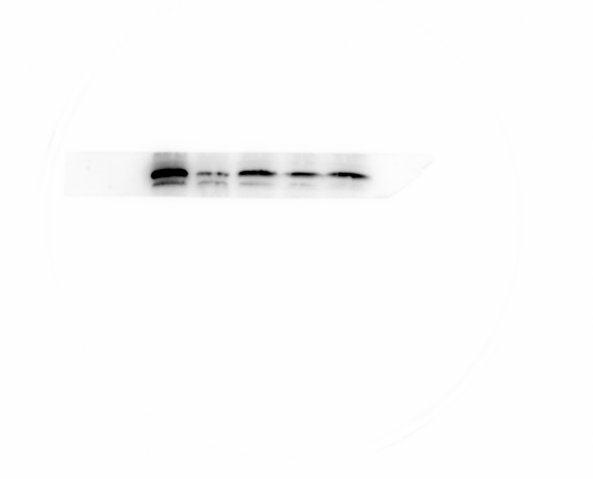


Fig S29 Original image of Bcl-2 in FIGURE 9E


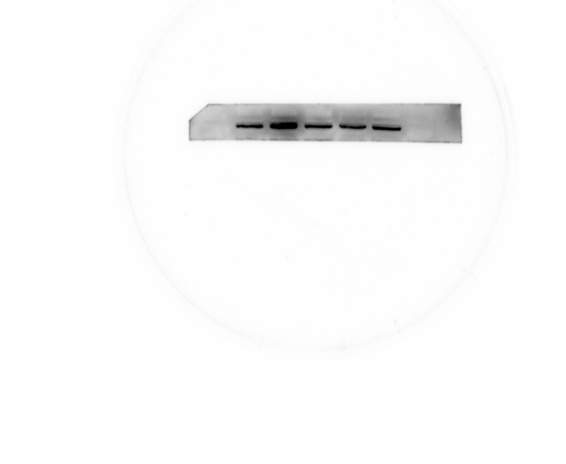


Fig S30 Original image of Bax in FIGURE 9E


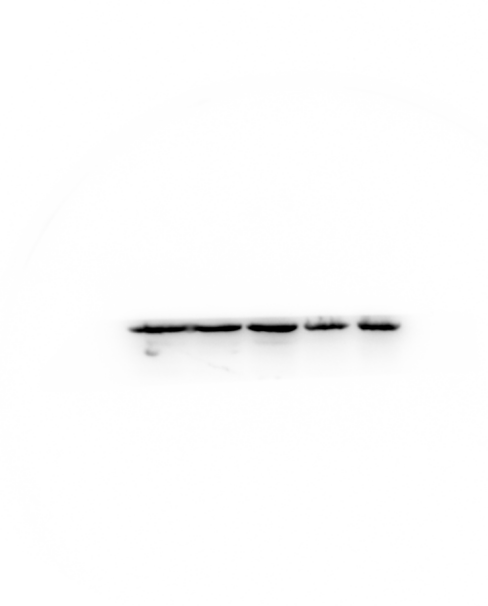


Fig S31 Original image of GAPDH in FIGURE 9E





Fig S32 Original image of P38 in FIGURE 9G


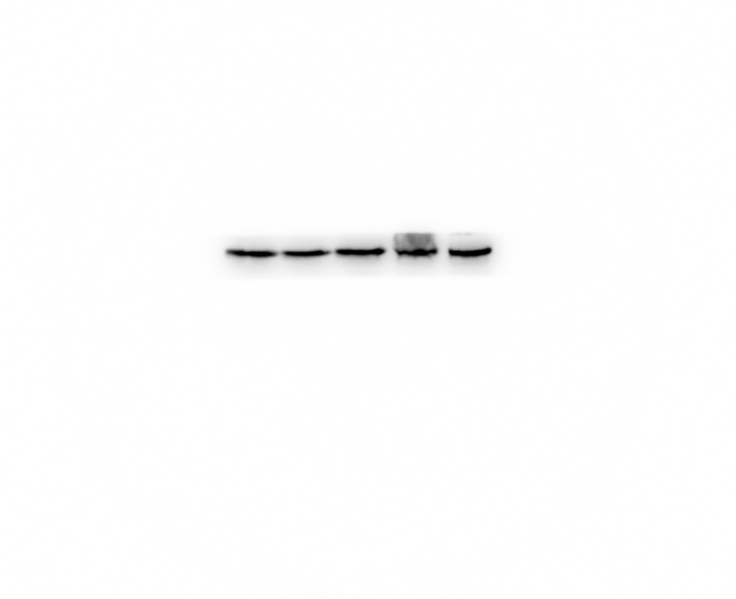


Fig S33 Original image of GAPDH in FIGURE 9G
